# Diagnosing Septate Uterus Using Three-Dimensional Ultrasound Using Three Different Classifications: An Interobserver and Intraobserver Agreement Study

**DOI:** 10.1055/s-0041-1740271

**Published:** 2021-12-21

**Authors:** Carla Peixoto, Maite Castro, Isabel Carriles, Maria de Arriba, Victoria Lapresa, Juan Luis Alcazar

**Affiliations:** 1 Department of Obstetrics and Gynecology, Centro Hospitalar São João, Porto, Portugal; 2 Faculty of Medicine, University of Porto, Porto, Portugal; 3 Centro de Infertilidad y Reproduccion Humana, Barcelona, Spain; 4 Department of Obstetrics and Gynecology, Clínica Universidad de Navarra, University of Navarra, Pamplona, Spain; 5 Department of Obstetrics and Gynecology, University and Polytechnic Hospital La Fe, Valencia, Spain; 6 Department of Obstetrics and Gynecology, University Hospital, Salamanca, Spain

**Keywords:** uterus, ultrasonography, congenital anomaly, útero, ecografia, anomalia congênita

## Abstract

**Objective** Currently, there are up to three different classifications for diagnosing septate uterus. The interobserver agreement among them has been poorly assessed.

**Objective** To assess the interobserver agreement of nonexpert sonographers for classifying septate uterus using the European Society of Human Reproduction and Embryology/European Society for Gynaecological Endoscopy (ESHRE/ESGE), American Society for Reproductive Medicine (ASRM), and Congenital Uterine Malformations by Experts (CUME) classifications.

**Methods** A total of 50 three-dimensional (3D) volumes of a nonconsecutive series of women with suspected uterine malformation were used. Two nonexpert examiners evaluated a single 3D volume of the uterus of each woman, blinded to each other. The following measurements were performed: indentation depth, indentation angle, uterine fundal wall thickness, external fundal indentation, and indentation-to-wall-thickness (I:WT) ratio. Each observer had to assign a diagnosis in each case, according to the three classification systems (ESHRE/ESGE, ASRM, and CUME). The interobserver agreement regarding the ESHRE/ESGE, ASRM, and CUME classifications was assessed using the Cohen weighted kappa index (k). Agreement regarding the three classifications (ASRM versus ESHRE/ESGE, ASRM versus CUME, ESHRE/ESGE versus CUME) was also assessed.

**Results** The interobserver agreement between the 2 nonexpert examiners was good for the ESHRE/ESGE (k = 0.74; 95% confidence interval [CI]: 0.55–0.92) and very good for the ASRM and CUME classification systems (k = 0.95; 95%CI: 0.86–1.00; and k = 0.91; 95%CI: 0.79–1.00, respectively). Agreement between the ESHRE/ESGE and ASRM classifications was moderate for both examiners. Agreement between the ESHRE/ESGE and CUME classifications was moderate for examiner 1 and good for examiner 2. Agreement between the ASRM and CUME classifications was good for both examiners.

**Conclusion** The three classifications have good (ESHRE/ESGE) or very good (ASRM and CUME) interobserver agreement. Agreement between the ASRM and CUME classifications was higher than that for the ESHRE/ESGE and ASRM and ESHRE/ESGE and CUME classifications.

## Resumo

**Objetivo** Atualmente, existem até três classificações diferentes para o diagnóstico de útero septado. A concordância interobservador entre eles tem sido pouco avaliada.

**Objetivo** Avaliar a concordância interobservador de ecografistas não especialistas para classificar úteros septados usando as classificações European Society of Human Reproduction and Embryology/European Society for Gynaecological Endoscopy (ESHRE/ESGE, na sigla em inglês), American Society for Reproductive Medicine (ASRM, na sigla em inglês) e Congenital Uterine Malformations by Experts (CUME, na sigla em inglês).

**Métodos** Foram utilizados 50 volumes tridimensionais (3D) de uma série não consecutiva de mulheres com suspeita de malformação uterina. Dois examinadores não especialistas avaliaram um único volume 3D do útero de cada mulher, mutuamente cegos. As seguintes medidas foram aferidas: profundidade de indentação, ângulo de indentação, espessura da parede do fundo uterino, indentação externa do fundo e relação entre indentação e a espessura da parede (I:WT, na sigla em inglês). Cada observador teve que atribuir um diagnóstico em cada caso, de acordo com os três sistemas de classificação (ESHRE/ESGE, ASRM e CUME). A concordância interobservador em relação às classificações ESHRE/ESGE, ASRM e CUME foi avaliada usando o índice kappa ponderado de Cohen (k). A concordância em relação às três classificações (ASRM *versus* ESHRE-ESGE, ASRM *versus* CUME e ESHRE-ESGE *versus* CUME) também foi avaliada.

**Resultados** A concordância interobservador entre os 2 examinadores não especialistas foi boa para a classificação ESHRE/ESGE (k = 0,74, intervalo de confiança [IC] 95%: 0,55–0,92) e muito boa para os sistemas de classificação ASRM e CUME (k = 0,95; IC 95%: 0,86–1,00; e k = 0,91; IC95%: 0,79–1,00, respectivamente). A concordância entre as classificações ESHRE/ESGE e ASRM foi moderada para ambos os examinadores. A concordância entre as classificações ESHRE/ESGE e CUME foi moderada para o examinador 1 e boa para o examinador 2. A concordância entre as classificações ASRM e CUME foi boa para ambos os examinadores.

**Conclusão** As três classificações apresentam concordância interobservador boa (ESHRE/ESGE) ou muito boa (ASRM e CUME). A concordância entre as classificações ASRM e CUME foi maior do que entre as classificações ESHRE/ESGE e ASRM e ESHRE/ESGE e CUME.

## Introduction

Congenital uterine malformations were described for the first time in 1800s and, since then, several classification systems have been developed for describing different types of uterine and cervical/vaginal anomalies, 1 whose incidence has been reported as of between 0.2 and 0.4% in the general population and of between 3 and 13% in infertile patients. 2 3 4 5 6 7

Classically, septate uterus has been associated with poor reproductive and obstetric outcomes, and surgical metroplasty is advocated in these cases, with the aim of improving these outcomes. 8 9 10 11 Notwithstanding, evidence that this surgery is beneficial is rather arguable. 12 Differently from septate uterus, arcuate/normal and bicornuate uteri do not require surgery. 8 9 10 11 However, from the beginning, there was some difficulty in the classification of uterine malformations, mainly due to the discrepancy between the diagnostic criteria and the diagnostic techniques used. 13 To overcome these limitations, three-dimensional (3D) ultrasound has been proposed as the gold standard technique to classify uterine malformations, as it seems to be better to evaluate the level of distortion of the uterine fundus, and also to reduce the interobserver variability. 14 15

The European Society of Human Reproduction and Embryology/European Society for Gynaecological Endoscopy (ESHRE/ESGE) and the American Society for Reproductive Medicine (ASRM) have both published their recommendations on how to classify uterine anomalies, using the coronal plane of the uterus. The ESHRE/ESGE classification suggests using an indentation-to-wall-thickness (I:WT) ratio > 50% for diagnosing a septate uterus and an external fundal indentation > 50% to diagnose a bicornuate uterus. 13 16 The ASRM classification considers a uterus as septate when there is both an indentation depth > 15mm and an indentation angle < 90°; a normal/arcuate uterus when there is both an indentation depth < 10mm and an indentation angle > 90°; and a bicornuate uterus when the external fundal indentation is > 10 mm. According to this classification, some cases could not be classified as septate or not-septate (falling in the so-called gray zone). 8 Although both classifications have very objective criteria, they do not coincide, which means that a high percentage of uteri classified as septate by the ESHRE/ESGE classification are classified as arcuate/normal by the ASRM classification. 17 18 19 20 More recently, a group of experts (Congenital Uterine Malformations by Experts [CUME]) proposed new criteria for diagnosing a septate uterus: indentation depth ≥ 10 mm, indentation angle < 140°, and I:WT > 110%. 18

The main objective of the present study was to assess the interobserver agreement of nonexpert sonographers in classifying septate uteri using the ESHRE/ESGE, ASRM, and CUME classifications in each case. Secondly, we also aimed to compare the agreement for each examiner for diagnosing septate uterus between the three different classifications (ESHRE/ESGE, ASRM, and CUME).

## Methods

The present study was a single-center retrospective analysis of patients with suspicion of congenital uterine malformation who underwent transvaginal ultrasound at the Department of Obstetrics and Gynecology of the Clínica Universidad de Navarra, Pamplona, Spain. Due to the study design and to the anonymization of the 3D volumes, formal approval by the Institutional Review Board from the Clínica Universidad de Navarra was waived. However, all women had given oral informed consent to acquire and use their 3D datasets for the present research. The present study was performed at the Clínica Universidad de Navarra between September and October 2018.

The inclusion criterion was: women with suspected uterine malformation in infertility setting who underwent 3D uterine evaluation. The exclusion criteria were: diagnosis of bicornuate or didelfis uterus or poor-quality 3D volume.

An expert examiner (Alcazar J. L.) randomly selected cases from the hospital database. Two nonexpert examiners (Peixoto C. and Castro M) evaluated a single 3D volume of the uterus of each woman. All 3D datasets had been acquired by one expert examiner (Alcazar J. L.) using either a Voluson 730 Expert or Voluson E8 machines (GE Healthcare, Chicago, IL, USA).

The nonexpert examiners had basic training on ultrasound in gynecology, with no special focus on uterine malformations, but both were undergoing a training program for ultrasound assessment of congenital uterine anomalies. Before the study, the nonexpert examiners took a short (2 hours) theoretical training session focused on the ESHRE/ESGE, ASRM and CUME classifications. Additionally, they read the original papers in which the criteria to classify uterine malformations were described. 8 13 18 They were also trained to use the 4D View Ultrasound software (GE Healthcare, Chicago, IL, USA).

**Fig. 1:**
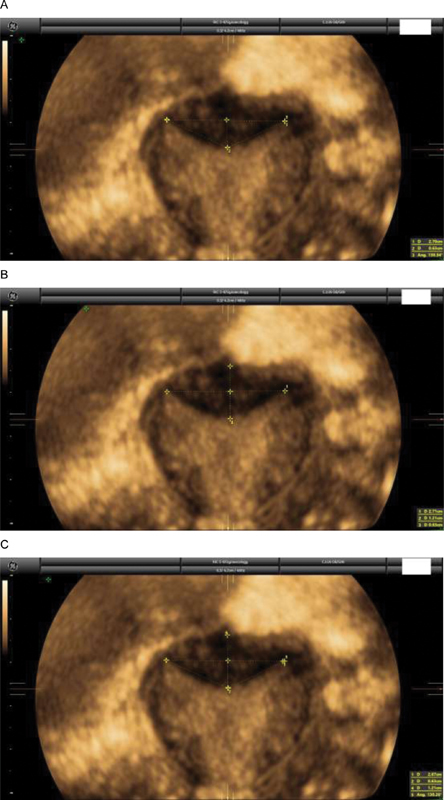
A case of a septate uterus according to the ESHRE/ESGE classification (I:WT = 52%) ( **A** ), but normal/arcuate according to the ASRM (indentation: 0.63 cm, angle: 130°) ( **B** ) and to the CUME (indentation: 0.63 cm, angle: 130°, I:WT: 52%) classifications ( **C** ).

The two observers manipulated the uterine 3D volumes, blinded to each other. After obtaining the coronal plane and using the Volume Contrast Imaging (VCI) function according to the CUME recommendations, 18 they performed the following measurements: indentation depth, indentation angle, uterine fundal wall thickness, external fundal indentation, and I:WT ratio. Each observer had to assign a diagnosis (normal/arcuate, septate) in each case, according to the three classification systems (ESHRE/ESGE, ASRM, and CUME) ( Fig. 1 ).

Arbitrarily, to avoid cases from falling in the grey zone, we decided the following: for the ASRM classification, in case that only one criterion was present, the case was considered as normal. For the CUME classification, the uterus was considered as septate if at least two criteria were present.

The examiners were also instructed not to discuss their impressions among themselves or with the expert after the assessment. We did not set a maximum time for performing evaluations of the 3D volumes.

The interobserver agreement between the two nonexpert examiners regarding the ESHRE/ESGE, ASRM, and CUME classifications was assessed using the Cohen weighted kappa index (k) with 95% confidence intervals (CIs) and percentage of agreement. 21

We also assessed the interobserver agreement for the two nonexpert examiners regarding the three classifications (ASRM versus ESHRE/ESGE, ASRM versus CUME, and ESHRE/ESGE versus CUME).

The kappa value was interpreted regarding the reporting of the reliability/strength of agreement as follows: poor < 0.20; fair = 0.21 to 0.40; moderate = 0.41 to 0.60; good = 0.61 to 0.80; and very good = 0.81 to 1.00. 22

Statistical calculations were done using GraphPad software (GraphPad Software, Inc., San Diego, CA, USA). Sample size calculation was not performed.

## Results

Forty-seven 3D volumes of women were included in the present study. This number was chosen arbitrarily. The interobserver agreement between the two nonexpert examiners for classifying uterine malformations is shown in Tables 1 , 2 and 3 . Overall, it was good for the ESHRE/ESGE (k = 0.74; 95%CI: 0.55–0.92) classification ( Table 1 ) and very good for the ASRM and CUME classifications (k = 0.96; 95%CI: 0.88–1.00; and k = 0.91; 95%CI: 0.79–1.00, respectively) ( Tables 2 and 3 ).

**Table 1:** Interobserver agreement for nonexpert examiners for classifying uterine congenital anomalies using the ESHRE/ESGE classification

ESHRE/ESGE						
		Examiner 1				
**Examiner 2**		**Normal/arcuate**	**Septate**	**Bicornuate**	**Weighted Kappa** **(95% CI)**	**Agreement** **(%)**
	Normal/arcuate	16	3	–	0.74 (0.55–0.92)	86%
	Septate	3	25	–		
	Bicornuate	–	1	2		

Abbreviations: CI, confidence interval; ESHRE, European Society of Human Reproduction and Embryology; ESGE, European Society for Gynaecological Endoscopy.

**Table 2:** Interobserver agreement for nonexpert examiners for classifying uterine congenital anomalies using the ASRM classification

ASRM						
		Examiner 1				
**Examiner 2**		**Normal/arcuate**	**Septate**	**Bicornuate**	**Weighted Kappa** **(95% CI)**	**Agreement** **(%)**
	Normal/arcuate	33	–	–	0.96 (0.88–1.00)	98%
	Septate	–	15	–		
	Bicornuate	–	1	1		

Abbreviations: CI, confidence interval; ASRM, American Society for Reproductive Medicine.

**Table 3:** Interobserver agreement for nonexpert examiners for classifying uterine congenital anomalies using the CUME classification

CUME					
		Examiner 1			
**Examiner 2**		**Normal/arcuate**	**Septate**	**Kappa** **(95%CI)**	**Agreement** **(%)**
	Normal/arcuate	27	1	0.91 (0.79–1.00)	96%
	Septate	1	18		

Abbreviations: CI, confidence interval; CUME, Congenital Uterine Malformations by Experts.

The agreement between the different classifications systems is shown in Tables 4 , 5 , 6 , 7 , 8 , 9 . When comparing the agreement for classifying uterine anomalies between the ESHRE/ESGE and ASRM classifications, we observed that it was moderate for both examiners ( Tables 4 and 5 ). We also observed that 14 cases were classified as septate by the ESHRE/ESGE classification and as normal/arcuate by the ASRM classification by both examiners. For both examiners, 9 of these cases were classified as normal/arcuate (64.3%) and 5 as septate (35.7%) when using the CUME classification.

**Table 4:** Intraobserver agreement for examiner 1 when using the ASRM and the ESHRE/ESGE classifications

Examiner 1						
		ASRM				
**ESHRE/ESGE**		**Normal/arcuate**	**Septate**	**Bicornuate**	**Weighted Kappa** **(95% CI)**	**Agreement** **(%)**
	Normal/arcuate	19	–	–	0.48 (0.28–0.68)	70%
	Septate	14	14	–		
	Bicornuate	–	1	2		

Abbreviations: ASRM, American Society for Reproductive Medicine; CI, confidence interval; ESHRE, European Society of Human Reproduction and Embryology; ESGE, European Society for Gynaecological Endoscopy.

**Table 5:** Intraobserver agreement for examiner 2 when using the ASRM and the ESHRE/ESGE classifications

Examiner 2						
		ASRM				
ESHRE/ESGE		Normal/arcuate	Septate	Bicornuate	Weighted Kappa (95% CI)	Agreement (%)
	Normal/arcuate	19	–	–	0.47 (0.27–0.67)	70%
	Septate	14	15	–		
	Bicornuate	–	1	1		

Abbreviations: ASRM, American Society for Reproductive Medicine; CI, confidence interval; ESHRE, European Society of Human Reproduction and Embryology; ESGE, European Society for Gynaecological Endoscopy.

**Table 6:** Intraobserver agreement for examiner 1 when using the CUME and the ESHRE/ESGE classifications

Examiner 1					
		ESHRE/ESGE			
**CUME**		**Normal/arcuate**	**Septate**	**Kappa** **(95%CI)**	**Agreement** **(%)**
	Normal/arcuate	18	10	0.59 (0.39–0.80)	79%
	Septate	–	19		

Abbreviations: CI, confidence interval; CUME, Congenital Uterine Malformations by Experts; ESHRE, European Society of Human Reproduction and Embryology; ESGE, European Society for Gynaecological Endoscopy.

**Table 7:** Intraobserver agreement for examiner 2 when using the CUME and the ESHRE/ESGE classifications

Examiner 2					
		ESHRE/ESGE			
**CUME**		**Normal/arcuate**	**Septate**	**Kappa** **(95%CI)**	**Agreement** **(%)**
	Normal/arcuate	19	9	0.63 (0.43–0.83)	81%
	Septate	–	19		

Abbreviations: CI, confidence interval; CUME, Congenital Uterine Malformations by Experts; ESHRE, European Society of Human Reproduction and Embryology; ESGE, European Society for Gynaecological Endoscopy.

**Table 8:** Intraobserver agreement for examiner 1 when using the CUME and the ASRM classifications

Examiner 1					
		ASRM			
**CUME**		**Normal/arcuate**	**Septate**	**Kappa** **(95%CI)**	**Agreement** **(%)**
	Normal/arcuate	28	–	0.77 (0.58–0.96)	89%
	Septate	5	14		

Abbreviations: ASRM, American Society for Reproductive Medicine; CI, confidence interval. CUME, Congenital Uterine Malformations by Experts.

**Table 9:** Intraobserver agreement for examiner 2 when using the CUME and the ASRM classifications

Examiner 2					
		ASRM			
**CUME**		**Normal/arcuate**	**Septate**	**Kappa** **(95%CI)**	**Agreement** **(%)**
	Normal/arcuate	28	–	0.77 (0.58–0.96)	89%
	Septate	5	14		

Abbreviations: ASRM, American Society for Reproductive Medicine; CI, confidence interval; CUME, Congenital Uterine Malformations by Experts.

The agreement between the ESHRE/ESGE and CUME classifications was moderate for examiner 1 and good for examiner 2 ( Tables 6 and 7 ). Finally, the agreement between the ASRM and CUME classifications was good for both examiners ( Tables 8 and 9 ).

## Discussion

As far as we know, this is the first study to assess the interobserver agreement of the three existing classification systems to describe normal, arcuate, and septate uterus. We have shown that the evaluation of 3D volumes of uteri is reproducible among nonexpert examiners.

The agreement between observers is higher when using the ASRM and CUME classifications. Actually, our data confirm the results previously reported by Ludwin et al., 20 who showed that the ASRM classification was better than the ESHRE/ESGE classification for diagnosing septate uterus. In addition, we have also shown that the criteria used by the new classification system (CUME), despite being apparently more complex, are highly reproducible among examiners (k = 0.91). This is an important finding, given that this new classification has not yet been validated after its publication.

In our study, the agreement between the ESHRE/ESGE and ASRM criteria was moderate. This finding is in line with those of previous studies 18 20 and raises concern regarding the use of the ESHRE/ESGE classification, since its use could lead to an overdiagnosis of septate uterus and to a potential increase of surgical corrections. 17 This is relevant since recent evidence suggests no benefit in obstetrical outcomes with surgery. 23 Our data also support the results published by the CUME group, 18 given that we demonstrated that, in comparison with CUME criteria, the ESHRE/ESGE classification overestimates the number of septate uteri. Overall, the agreement between the CUME and the ESHRE/ESGE and the ASRM classifications was good, but it was slightly better between the CUME and ASRM classifications than between the CUME and ESHRE/ESGE classifications.

An interesting question is related to the fact that if the ESHRE/ESGE classification would use the I:I + WT ratio, instead of the I:WT ratio, the rate of septate uterus would be similar to CUME classification.

The strengths of the present study are its design and the use of an optimal diagnostic method (3D ultrasonography) for diagnosing uterine anomalies. 6 24 The participation of nonexpert examiners could be seen as a potential strength, since it allows the evaluation of the reproducibility of the different classifications in “everyday practice.”

However, certainly, our study design can be also considered as a limitation, since the sources of variability regarding the real-time ultrasound and 3D volume acquisition were not taken to account, since the two observers have used previously acquired 3D datasets, which may overestimate the reproducibility of the measurements.

As stated above, our study has limitations. One limitation of the present study is that the examiners had to manipulate the 3D volumes by rotation in all 3 orthogonal planes. This manipulation has an inherent variability between observers, 18 20 as the same uterus might provide different images depending on the angle at which the coronal plane is obtained. Other possible limitations of the present study are the small number of cases analyzed and the high quality of 3D volumes, which may have contributed to a lower number of “discrepant” cases. One final limitation that must be mentioned is that we arbitrarily decided to assume that there were no gray-zone cases, since the uteri were classified as septate only when both criteria of the ASRM classification or at least two criteria of the CUME classification were present. It is clear that this point could bias the results, since, somehow, we forced providing a diagnosis in all cases, which is not true in the case of the ASRM classification.

Despite these limitations, we consider that our findings may have clinical relevance and should prompt further studies to determine which classification should be used.

## Conclusion

In general, the three classifications have good (ESHRE/ESGE) or very good (ASRM, CUME) interobserver agreement, which makes them all good methods to classify congenital uterine anomalies. However, agreement between the ASRM and the CUME classifications was higher than that for the ESHRE-ESGE and the ASRM and for the ESHRE/ESGE and the CUME classifications.
